# Supplementary figures and images for: Tumor cell intrinsic and extrinsic features predict prognosis in estrogen receptor positive breast cancer
Source: PLoS Comput Biol. 2022 Mar 9;18(3):e1009495. doi: 10.1371/journal.pcbi.1009495 (PMC8936467; doi:10.1371/journal.pcbi.1009495)

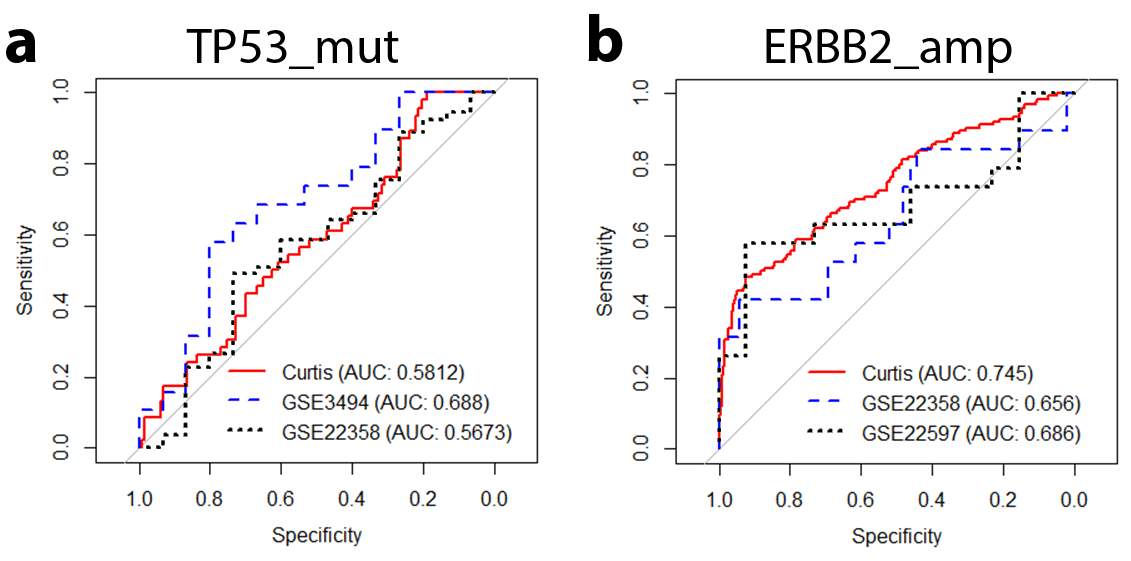

Supplement: S1 Fig — ROC curves showing that TP53_mut (a) and ERBB2_amp (b) predict driver aberration status at relatively low AUC scores in the ER negative breast cancer patients as compared to the ER positive patients. (TIF) [file pcbi.1009495.s001.tif]

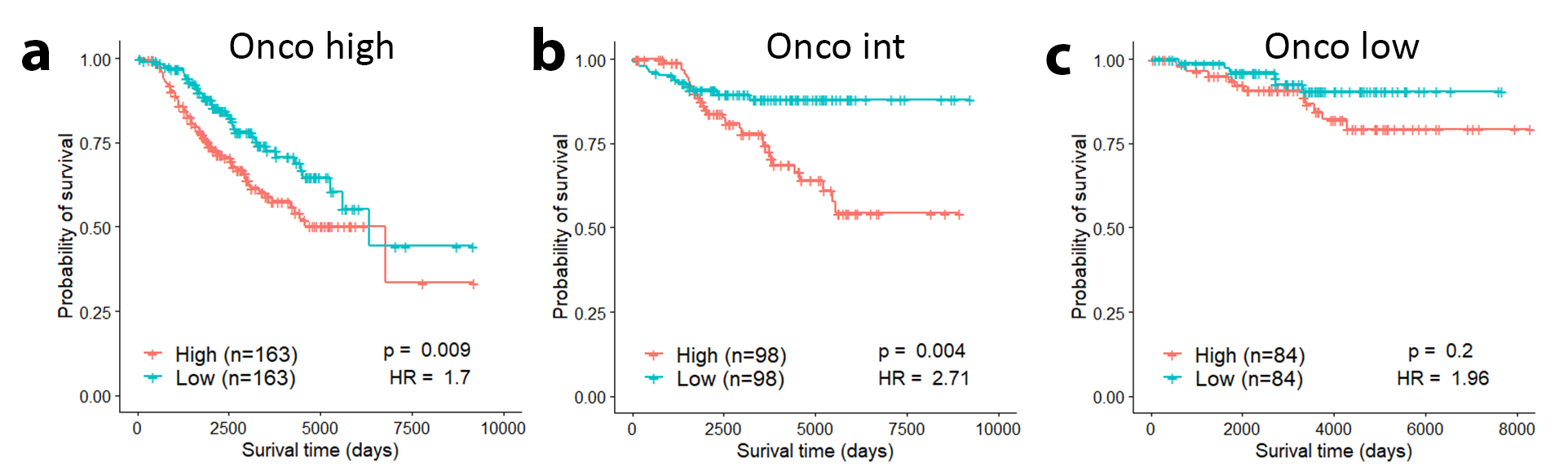

Supplement: S2 Fig — Our optimized model’s risk prediction without including clinical features is able to significantly stratify the Onco high (a) and intermediate (b) risk classes, and moderately stratifies the Onco low (c) risk class. (TIF) [file pcbi.1009495.s002.tif]
