# Supplementary material for: Tumor cell intrinsic and extrinsic features predict prognosis in estrogen receptor positive breast cancer
Source: PLoS Comput Biol. 2022 Mar 9;18(3):e1009495. doi: 10.1371/journal.pcbi.1009495 (PMC8936467; doi:10.1371/journal.pcbi.1009495)
Supplement: Attachment — Submitted filename: plosCB_ResponseLetter_Chengedit.docx [file pcbi.1009495.s007.docx]

### Response Letter

### Reviewer 1:

### -- Ref1.0 – Overall comments 1--

| Reviewer  Comment | Yao et al described their work to address the heterogeneity in the prognosis in ER+ breast cancer using both genomic mutations within the tumor cells, and also extrinsic features in the tumor microenvironment. The study was based on TCGA and multiple datasets from METABRIC and GEO, and identified 72 genomic aberrations and 6 immunological features for the model development. The model was able to reach better performance than the traditional Oncotype DX assay. Overall, the study was well performed. |
| --- | --- |
| Author  Response | We thank the review for the comments. Below are our point by point responses to the reviewer’s comments. |
| Excerpt From  Revised Manuscript |  |

### -- Ref1.1 – Immunological features benefit --

| Reviewer  Comment | As mentioned by the authors, adding imm features does not seem to improve the prediction model (0.817 vs 0.811, Figure 3e). Can authors comment on the additional benefit of including immunological features? |
| --- | --- |
| Author  Response | We thank the reviewer for this reasonable comment. Possibly because breast cancer is relatively immune cold compared to other cancers, we did not observe a significant advantage in including them in our full integrated models. In other cancers, immune features may be more important in prediction. However, we also note that since we have 72 intrinsic genomic features versus only 6 immune features, it is likely that the 72 genomic features already encapsulate the information provided by the immune features. Indeed, some of the signatures (e.g., TP53 mutation and CDH1 mutation) are significantly correlated with immune features. Even so, the memory B immune infiltration score was included in the random forest optimized model. Thus, the advantage of including the immune features would be that they allow for better and more accurate feature optimization of the predictive model. In addition, they also provide critical insights about different immunological features in terms of prognostic prediction. |
| Excerpt From  Revised Manuscript | We included the following sentences on page 16 in the revised manuscript: “Even so, the memory B immune infiltration score was included in the random forest optimized model. Including immune features may allow for better and more accurate feature optimization of the predictive model. In addition, they also provide critical insights about different immunological features in terms of prognostic prediction.” |

### -- Ref1.2 – Accuracy of models in abstract--

| Reviewer  Comment | In the abstract, the authors should report the actual accuracy of different models for comparison. |
| --- | --- |
| Author  Response | We thank the reviewer for this comment. We have included the AUC of the optimized random forest model to compare with that of the Oncotype DX model |
| Excerpt From  Revised Manuscript | In the abstract, we added the following sentences: “(our optimized random forest model AUC=0.841, Oncotype DX model AUC=0.792, p=0.04).” |

### -- Ref1.3 – Significance test for AUC values--

| Reviewer  Comment | The authors indicated that accuracy of the optimized model was much higher than the Oco+Clin model and the Clin model. It would be useful to report if this difference is significant or not. |
| --- | --- |
| Author  Response | We thank the reviewer for this suggestion. We have followed the reviewer’s suggestion and performed Delong’s test for two correlated ROC curves to test our hypothesis that the AUC of the optimized model is higher than that of the Onco+Clin model and the Clin model. We found that the optimized model AUC is indeed significantly higher than that of the Onco+Clin model (p=0.04) and the Clin model (p=2e-4). |
| Excerpt From  Revised Manuscript | In the abstract, we added the p value: “(our optimized random forest model AUC=0.841, Oncotype DX model AUC=0.792, p=0.04).”  On page 11, we added details on the p value and its calculation: “The accuracy of this optimized model was AUC=0.841 according to cross-validation results in the Curtis data, which was much higher the Onco+Clin model (AUC=0.791, p=0.04) and the Clin model (AUC=0.763, p=2e-4) (Fig. 4b). The significance test was performed using Delong’s test for two correlated ROC curves.” |

### -- Ref1.4 – Relative risk in Cox model--

| Reviewer  Comment | In page 13, “In particular, patients in the high risk category had a hazard 3.196 times that of the intermediate risk category (p = 3e-11), and patients in the intermediate risk category had a hazard 2.523 times that of the low risk category (p = 0.001)”. It would be useful to use one group as the referent, and report the relative risk in the other two groups. |
| --- | --- |
| Author  Response | We thank the reviewer for this comment. We have followed the reviewer’s suggestion and used the low risk category as the referent and reported the relative risk in the intermediate and high risk categories. We also edited Figure 6 to account for this change. |
| Excerpt From  Revised Manuscript | In page 13, we changed all references of high risk to intermediate risk to high risk to low risk: “In particular, patients in the high risk category had a hazard 8.015 times that of the low risk category (p = 6e-20)… Patients in the high risk group had a hazard 7.394 times that of the low risk category (p = 3e-17)… Patients classified as high risk had a hazard 3.533 times that of patients in the low risk category (p = 2e-7).” |

### -- Ref1.5 – Description of Curtis and Ur-Rehman dataset in results--

| Reviewer  Comment | Please provide some brief description of the Curtis dataset and Ur-Rehman dataset in the Results section because the detailed information of both datasets were provided in later sections. |
| --- | --- |
| Author  Response | We thank the reviewer for this comment. We have included a brief description of the datasets in the results section. |
| Excerpt From  Revised Manuscript | In the results, we added the following sentences on page 9: “dataset, which contains microarray data for 1508 ER+ breast cancer patients [39]”  On page 12, we added: “the Ur-Rehman dataset, which contains curated data from 856 ER+ breast cancer patients [52]” |

### -- Ref1.6 – More details on feature names--

| Reviewer  Comment | Please provide more informative description of feature names in the main text and Figures. For example, MemB is for Memory B gene expression profile. |
| --- | --- |
| Author  Response | We thank the reviewer for this comment. We have defined the abbreviations for the immune cell infiltration scores at the first place of use. |
| Excerpt From  Revised Manuscript | On pages 9-10, we added the following definitions: “Naïve B (NavB), Memory B (MemB), CD4+ T (CD4T), CD8+ T (CD8T), natural killer cells (NKcell), and Monocytes” |

### -- Ref1.7 – Definition of abbreviations --

| Reviewer  Comment | Please define CI, ROC and AUC at the first place to use |
| --- | --- |
| Author  Response | We thank the reviewer for this comment. We have included the definition of CI, ROC, and AUC at the first place of use. |
| Excerpt From  Revised Manuscript | On page 13, we added the following definition: “concordance index (CI)”  On page 8, we added the following definition: “receiver operating characteristic (ROC)”  On pages 8-9, we added the following definition: “area under the receiver operating characteristic curve (AUC)” |

### Reviewer 2:

### -- Ref2.0 – Overall comments --

| Reviewer  Comment | In this study, the authors suggested that the prognosis of ER+ breast cancer patients can be inferred by both tumor-intrinsic (genomic aberrations) and extrinsic (immunological) features. They proposed a systematic framework that utilizes gene expression data for feature extraction and predicts prognosis of ER+ breast cancer patients. Based on TCGA data, 72 genes for tumor-intrinsic score, and 6 immune cell types for infiltration score were selected. Compared to Oncotype Dx, the most widely validated method for predicting prognosis for ER+ breast cancer patients, their framework showed the improved prognostic performance on external validation dataset. Further, they trimmed features to optimize their models and determined important features for predicting prognosis. This paper analyzed the prediction results extensively. |
| --- | --- |
| Author  Response | We thank the reviewer for carefully reviewing our manuscript and providing us with valuable comments and suggestions. All the comments are reasonable. The following are our point-by-point responses. |
| Excerpt From  Revised Manuscript |  |

### -- Ref2.1 – More details on score calculation --

| Reviewer  Comment | More detailed explanation about how to calculate a sample-specific score based on gene signature, and the extrinsic score based on immunological profiles should be added to the Method section, although the authors used methods developed by others. It seems that too many steps were omitted, compared to the Definition of weighted gene signatures for genomic events section. |
| --- | --- |
| Author  Response | The reviewer made a good suggestion. We have included more details on the calculation of sample specific intrinsic and extrinsic scores. |
| Excerpt From  Revised Manuscript | We added the following sentences in the methods section on page 20: “First, we use median normalization on the gene expression profile. We then rank the expression profile in order of decreasing expression, **g** = {g_1_, g_2_, … g_n_}, where n is the total number of genes. Then, we calculate a foreground f(i) and background function b(i) as shown below.  $f\left( i \right)= \frac{\sum_{k=1}^{i} g_{k}w_{k}}{\sum_{k=1}^{n} g_{k}w_{k}},1\leq i\leq n$  $b\left( i \right)= \frac{\sum_{k=1}^{i} g_{k}({1-w}_{k})}{\sum_{k=1}^{n} g_{k}({1-w}_{k})},1\leq i\leq n$  The foreground function captures the distribution of the highly informative genes, whereas the background function captures random distribution. The maximum deviation between these functions is then calculated. For the w^+^ weights, the score is defined Score^+^, and for the w^-^ weights the score is defined Score^-^. We then normalized the Score^+^ and Score^-^ scores by dividing them by their null distribution. The null distribution is calculated based on recalculating the Score^+^ and Score^-^ scores, but using a random ordering of the gene expression profile **g**. This process is permuted 1000 times to generate the null distribution. The sample specific score for each genomic aberration is finally calculated as the difference between the normalized Score^+^ and Score^-^.” |

### -- Ref2.2 – Clarify inclusion of clinical variables --

| Reviewer  Comment | In the Integrative models for classifying good and poor prognosis patient groups section, in Fig. 3f, and Supplementary Table 3, there were no clinical variables while the authors mentioned that the Sig + Imm + Clin RF model were used to determine which variables were significant. Make it clear. |
| --- | --- |
| Author  Response | The reviewer made a good catch. In Fig. 3f, clinical variables are included, as stage is shown as a label. However, in Suppl. Table 3, the clinical variables had been omitted. We have replaced Suppl. Table 3 with the clinical variables added to maintain consistency that clinical variables are included. The variables are selected based on their prognostic significance reported in previous studies and availability from the relevant datasets. |
| Excerpt From  Revised Manuscript |  |

### -- Ref2.3 – Specify dataset --

| Reviewer  Comment | In the Integrative models for predicting prognostic risk based on Cox regression section, specify a dataset used for analyzing 1,494 (747 High + 747 Low) patients in Fig 5. |
| --- | --- |
| Author  Response | We thank the reviewer for this suggestion. In the revision, we have specified the dataset used for the Cox regression section in Fig. 5. All the analyses done in this section was done in the Curtis dataset. |
| Excerpt From  Revised Manuscript | On page 12, we added: “in the Curtis dataset” |

### -- Ref2.4 – Typos--

| Reviewer  Comment | Minor: 1. There are some typos. The followings are examples. The authors need to check the manuscript carefully. Page 7: [39]Results -> Results Page 17: An additional seven datasets => Additional seven datasets Page 32: distinguishes -> distinguish Page 34: In Figure 5, (d) is TNFRSF17_amp and (e) is MemB while MemB (d), and TNFRSF17_amp (e) in the legend. Make it clear. |
| --- | --- |
| Author  Response | We thank the reviewer for identifying these typos. We have addressed these typos in the revised manuscript. |
| Excerpt From  Revised Manuscript | Page 7: [39]Results -> Results Page 17: An additional seven datasets => Additional seven datasets Page 32: distinguishes -> distinguish Page 34: We have fixed the figure caption to be consistent with the Figure: “MemB (**d**), and TNFRSF17_amp (**e**)” to “TNFRSF17_amp (**d**), and MemB (**e**)” |
